# Supplementary material for: Small means immoral? The impact of spatial size metaphor on moral judgment
Source: PLoS One. 2025 May 27;20(5):e0324991. doi: 10.1371/journal.pone.0324991 (PMC12111654; doi:10.1371/journal.pone.0324991)
Supplement: Attachment — Submitted filename: Response to Reviewers.docx [file pone.0324991.s003.docx]

Dear editor:

We appreciate very much your insightful comments and critiques. These comments are very helpful for us to revise this paper. We have carried out a comprehensive revision on the manuscript in accordance to your comments. The revisions were addressed point by point below. For the convenience of review, I marked all the changes in red fonts in the revised manuscript.

Journal requirements:

When submitting your revision, we need you to address these additional requirements.

1. Please ensure that your manuscript meets PLOS ONE's style requirements, including those for file naming. The PLOS ONE style templates can be found at

https://journals.plos.org/plosone/s/file?id=wjVg/PLOSOne_formatting_sample_main_body.pdf and

https://journals.plos.org/plosone/s/file?id=ba62/PLOSOne_formatting_sample_title_authors_affiliations.pdf

Response: Thank you for your valuable comments. We have already made adjustments according to the template.

2. Please include your full ethics statement in the ‘Methods’ section of your manuscript file. In your statement, please include the full name of the IRB or ethics committee who approved or waived your study, as well as whether or not you obtained informed written or verbal consent. If consent was waived for your study, please include this information in your statement as well.

Response: Thank you for your valuable comments. We have already added the full name of ethics committee and the information of informed written consent from participants in Materials and Methods of Study 1. The changes as requested are as follows:

*All studies were approved by the Ethics Committee of Chongqing Normal University. All informed consent forms were signed before participants completed the questionnaire for evaluation and began the studies. All experimental procedures adhered to the ethical guidelines outlined in the Declaration of Helsinki.*

3. Please include captions for your Supporting Information files at the end of your manuscript, and update any in-text citations to match accordingly. Please see our Supporting Information guidelines for more information: http://journals.plos.org/plosone/s/supporting-information.

Response: Thank you for your valuable comments. This article does not have supporting information.

Review Comments to the Author

Reviewer #1: This paper describes three psychological experiments that seek to show a connection between moral concepts and physical space. One experiment examined a relationship between a large and small fonts used for moral and immoral words, another examined a relationship between moral and immoral words and larger and smaller squares, and the third examined how participants responded to moral dilemmas when in a bigger or smaller room. I think that the conceptual metaphor that “big is good” is already well-established, and so the paper would be stronger if it began with some explanation of what the paper contributes that is new. Nevertheless, the paper discusses an interesting and appropriate topic for PLOS One, and it could be published once revised. However, conceptual problems and grammar problems mean that the revisions would be many.

Response: Thank you for your valuable comments. We have revised the manuscript, providing more detailed explanations to address conceptual issues and further polishing the grammar with additional edits and refinements.

**Grammatical problems**
This paper has a significant number of grammar problems. I saw words mis-spelled, verb agreement problems, and sentences without punctuation. The paper speaks of “bodily size” instead of body size (p. 17). In several cases, the sentences are ungrammatical to the point that the paper could not be published unless they were corrected, but the problems do not obscure the meaning intended. Here are some examples of this.

Response: Thank you for your valuable comments. We have revised the vocabulary, grammar, and punctuation of the paper, and restructured complex sentences to ensure the key arguments remain unobscured.

1. The following sentence is missing its articles: “Source concept is direct experience in daily lives” (p. 2). This should be “A source concept is a direct experience in a person’s daily life” or, better, since a concept is not an experience, “A source concept is found in a direct experience in a person’s daily life.”

Response: Thank you for your valuable comments. The changes as requested are as follows:

*A source concept originates from a direct experience in a person's daily life, whereas a target concept is an abstract and vague idea, entity, or emotion [1].*

2. In some cases, the verbs are not idiomatic. For example, the paper says that Conceptual Metaphor Theory “believes” that understanding is such-and-such (p. 2). But a theory is not a person and so it cannot believe things. It would be better to say: According to Conceptual Metaphor Theory, understanding is such-and-such.

Response: Thank you for bringing it to our attention. The error has been corrected. This sentence has been modified:

*According to CMT, understanding target concepts is facilitated by drawing on well-known and clearly represented prior experiences (source concepts).*

3. The paper says that “Merleau-Ponty proposed that the importance of the body and senses arising within organizational behavior” (p. 3). However, this is not a complete sentence and so I am not sure what the authors are trying to say. I think that the authors meant to write that Merleau-Ponty wrote about the importance of the body and senses arising within organizational behavior.

Response: Thank you very much for pointing it out. The changes as requested are as follows:

*With the development of experiential philosophy and phenomenology, Merleau-Ponty [4] emphasized that the importance of the body and senses arises within organizational behavior.*

4. One experiment describes concepts “of two different sizes (8 cm x 8cm and 4 cm x cm)” (p. 10). I think that the two different sizes described here are actually squares, not concepts, and I imagine that the squares are made of paper, but here again the paper was hard to follow.

Response: Thank you for your advice. This has been added to the article and your help in improving it is much appreciated. The changes as requested are as follows:

*The target concepts included 10 moral and 10 immoral words, while the source concepts consisted of two squares of different sizes (8 cm x 8 cm and 4 cm x 4 cm). All materials were presented on a computer screen.*

However, the English is often ungrammatical to the point that the paper’s argument is obscured. Here are some examples of this.

1. The authors write that “the words in a bigger font gave a more positive meaning” (p. 3) but what they mean to say is the importantly different claim that the bigger font of the words gave a more positive meaning.

Response: Thank you for your valuable comments. This sentence has been modified:

*The larger font of the words conveyed a more positive meaning, illustrating the metaphorical association between 'big and positive' and 'small and negative".*

2. The paper says that certain scholars were “founding that participants would increase the perceived size of object” (p. 3). Setting aside the problem that the word “object” here is missing its article, the word “founding” does not make sense in this context. The authors probably mean something like “speculating.” However, perhaps they mean the very different idea that the cited paper “found” that participants did this, in the sense that the scholars discovered or showed it.

Response: We would like to apologize for our oversight. Thank you for bringing it to our attention. The error has been corrected. This sentence has been modified:

*Martijn, Henk, and Ruud [3] examined the desire to achieve the goal and found that participants would increase the perceived size of an object, which would help achieve the goal.*

3. The paper says this: “when the participant was deprived of the freedom to drink water, the subject would perceive a bigger cup size” (p. 3). But what the authors mean that the scholars hypothesized that when the participant was deprived of the freedom to drink water, the subject would perceive a bigger cup size. By failing to include the phrase “They hypothesized that,” the paper turns a hypothesis into a fact.

Response: Thank you for bringing it to our attention. We have already added “*the researchers hypothesized that*”. This sentence has been modified:

*For instance, the researchers hypothesized that when a participant was deprived of the freedom to drink water, they would perceive a cup as larger.*

4. I could not figure out what is meant by this sentence: “A priori power is consistent with the above” (p. 9). Of course, “a priori” is a Latin phrase that is important in philosophy, but I am not sure what is meant by “a priori power.” The paper is not making a philosophical argument, and it had never introduced the idea of “a priori power” or even the idea of power. Later, the paper says this: “A priori power analysis is consistent with the above” (p. 14). This makes me suspect that the word “analysis” was dropped from the first sentence, but I don’t know what “a priori power analysis” or even what “power analysis” is.

Response: Thank you for your advice. This has been added to the article and your help in improving it is much appreciated. The changes as requested are as follows:

*Study 1：According to calculation using G*power 3.1 [25], for the two-way repeated measures analysis of variance applicable to this study, with a significance level of α = 0.05, a large effect size (f = 0.4), and a predicted statistical power (1-β) of 0.8, the required total number of research subjects was 22.*

*Study 2：According to calculation using G*power 3.1 [25], for the Paired samples t-tests applicable to this study, with a significance level of α = 0.05, a large effect size (f = 0.8), and a predicted statistical power (1-β) of 0.8, the required total number of research subjects was 15.*

*Study 3：According to calculation using G*power 3.1 [25], for the independent samples t-test applicable to this study, with a significance level of α = 0.05, a large effect size (d= 0.8), and a predicted statistical power (1-β) of 0.8, the required total number of research subjects was 52.*

**Conceptual problems**
None of these conceptual problems is severe.

1. In the abstract, the authors claim that the paper shows that there is an unconscious reality between moral concepts and size (pp. 1, 4). I think that they do not mean (or, at least, they should not mean) that they have discovered a reality between moral concepts and size. I think that they mean to claim that there is a *relationship* between moral concepts and size.

Response: Thank you for your valuable comments. We have changed word to relationship. This sentence has been modified:

*Collectively, these three studies demonstrate that the unconscious psychological relationship between moral concepts and the spatial dimension of size influences individuals’ abstract moral judgments under embodied cognition.*

2. The paper says “In english expressions, there are still some words (such as large, wide, broad) to describe moral behavior” (p. 2). I was confused why the paper says that these expressions “still” exist, as if such expressions are old-fashioned or being eliminated. And though the claim is likely to be correct, I cannot think of any expressions for moral behavior with these terms, and so I wished that the paper had said what they were.
Response: Thank you for your valuable comments. We have removed 'still' from the sentence. This sentence has been modified:

*Similarly, in English, certain words such as large, wide, and broad are used to describe moral behavior, while words such as small, narrow, and thin are often associated with immoral behavior. People comprehend abstract concepts not only through concrete notions of size but also by recognizing the correlation between size and emotional valence.*

3. The describe a contrast between “self-purification” and “self-cleansing” (p. 3). These seem identical and I think that the problem is that the paper was written incorrectly.

Response: We would like to apologize for our oversight. Thank you for bringing it to our attention. We have changed "self-purification" to "self-dirtiness". This sentence has been modified:

*Within the framework of the purification metaphor, Ding and Wang [8] observed that participants primed with self-dirtiness exhibited heightened reactivity to negative moral emotion words compared to those primed with self-cleansing.*

Reviewer #2: The studies described in this article—particularly Study 3—warrant dissemination as they extend the knowledge we have about how humans make moral judgements, and how such judgments are embodied. However, the article is not currently written to make that contribution clear.

Response: Thank you very much for pointing it out. Interpretation of the introduction has been added as described below:

*These studies aim to expand knowledge on how humans make moral judgments and how these judgments are embodied. The anticipated contributions lie in bridging gaps between unconscious cognitive processes and embodied metaphor theory, potentially informing interdisciplinary discussions in moral psychology, linguistics, and environmental design.*

The first half of the literature review lacks organization and precision. It moves from similar studies designed to show the connection between size and morality, to general remarks about the place of embodiment (e.g. Merleau-Ponty, whose connection to embodied cognition needs to be nuanced since he predates it), to an assortment of embodied cognition studies. Within this section there is an elision between conceptual metaphor theory and embodied cognition—not all advocates of CMT advocate all theories of embodied cognition, and there are different theories of embodied cognition. Right now “embodied cognition” is defined without citation within the discussion on Merleau-Ponty. I don’t think a paper like this needs to necessarily wade into these theoretical waters, but if it does the section should be clearer and more developed.
Response: Thank you for your advice. This has been added to the article and your help in improving it is much appreciated. We made that all theories discussed in the article were presented within the framework of Embodied Cognition and add some sentences. Interpretation of the introduction has been added as described below:

*Within the framework of embodied cognition, Lakoff and Johnson [1] proposed the Conceptual Metaphor Theory (CMT), which suggests that the conceptual systems underlying human thought, cognition, and behavior are structured by metaphors.*

*Furthermore, conceptual representation, as a fundamental and primary cognitive ability of humans, is inherently linked to the individual's perceptual-motor system and cannot exist independently of it [5].*

*Embodied cognition theory holds that cognitive activities are not only inseparable from the brain but are also closely linked to the interaction between the body and the environment.*

More critically, the discussion of how the current studies build on this literature is underdeveloped. For example, in discussing Study 1 vis-à-vis Lu, Guo and Jiang (2017) the article states that “Nonetheless, the existence of an unconscious mental association with moral conceptual size-space metaphors has yet to be empirically substantiated, nor has their metaphorical consistency been rigorously examined within the context of complex, embodied moral judgment tasks. Consequently, study 1 verifies the psychological reality of the existence of metaphors between moral concepts and big and small spaces on an unconscious level…” but the study seems to be replicating Lu, Guo and Jiang. Multiple times in the article the difference is cached out in terms of unconscious vs conscious connections (see for example the end of the discussion of study 3) but it’s not clear how or why Studies 1 and 2 deal with the unconscious whereas studies like Lu, Guo, and Jiang (2017) deal with the conscious.

Response: We have made the revisions according to your suggestions. We sincerely appreciate your correction, which has enabled our manuscript to be improved. In the introduction section, Lakens, Semin, and Garrido (2011), Ding and Wang (2023), and Lu, Guo, and Jiang (2017) have been added to address their experimental procedure controls over response times. Additionally, the response time constraints (responses within 1500ms) implemented in both Studies 1 and 2 are now specified in both the introduction and procedure sections. The changes as requested are as follows:

Interpretation of the introduction has been added as described below:

*Regarding the experimental procedure, participants were required to provide an optimal rating for each word.*

*Additionally, in the experimental procedure, participants were not given a time limit to respond to the words, suggesting that they had ample time to process them thoroughly.*

*In their research procedure, participants were given 2500 ms to respond to each word. Most studies are conducted at the conscious level.*

Interpretation of each Study has been added as described below:

*Study 1: If a response was not made within 1500 milliseconds, the word automatically disappeared, followed by the reappearance of the red “+” cue for another 500 milliseconds to signal the next trial.*

*Study 2: (Button presses had to be completed within 1500 ms or the interface would advance to the next screen.)*

Study 3 is the most original, yet it is given the least explanation (this may be an organization problem, as I wonder if lines like the one I quoted in the paragraph above weren’t supposed to be specifically about Study 3, insofar as it specifically is dealing with spaces). Whereas the Stroop Test and the IAT have the subject engaging size depicted on screens, Study 3 examines the size of the space the subject is in. This difference seems particularly relevant given the authors’ interest in embodied cognition, as one could argue that the results are more suggestive of the brain’s hijacking of sensorimotor systems for moral reasoning (vs. merely “associating” size and morality). (And if this is what the authors’ mean by conscious vs. unconscious that needs to be better explained, and clarified that it is Study 3 that specifically offers this contribution).

Response: We have made the revisions according to your suggestions. We sincerely appreciate your correction, which has enabled our manuscript to be improved. And the changes as requested are as follows:

*The brain is embedded in the body, and the body is embedded in the environment, forming an integrated cognitive system [23]. Research has shown that individuals represent and process conceptual knowledge through simulations of sensorimotor experiences [24]. For instance, when processing the concept of "apple," the brain activates multimodal sensory information, including visual (red color), tactile (smooth texture), and gustatory (sweet taste) inputs, to construct an understanding of the concept. Consequently, Study 3 delves into the exploration of how size-space perceptions link to an individual’s moral judgments by unconsciously activating the participants' perceptual information.*

*Embodied cognition posits that the environment is an integral part of the cognitive system [39]. The ambiguous nature of the dilemmas was designed to trigger cognitive conflict, prompting participants to rely on environmental cues [28].*

There is another important consideration—whereas in Studies 1 and 2 it is clear that bigness and morality are being linked, it is not so clear once we are putting a subject in a room. Critically, a person in a big room may feel smaller; the screen that they are reading the moral dilemma off will also appear relatively smaller. So the design of Study 3 introduces some complexity that the authors need to address. In other words I don’t think it’s a straight of a line from seeing a big box on a screen to being in a big box.
Response: Thank you for your advice. As requested we have added sample adoption criteria, thank you for your help. Interpretation of the discussion has been added as described below:

*Schwartz, Tesse, and Powell (1982) found that an individual’s position in the power hierarchy is closely linked to their height and weight, with taller and stronger individuals often perceived as having higher power and social status. Gibbs et al. (2004) further clarified that this association arises from inherent physical attributes (e.g., height) rather than learned bodily experiences (e.g., physical training).*

*Embodied cognition posits that the environment is an integral part of the cognitive system [39]. The ambiguous nature of the dilemmas was designed to trigger cognitive conflict, prompting participants to rely on environmental cues [29]. Thus, in Study 3, although individuals may appear smaller in a large room, they tend to focus more on the environment’s vastness while overlooking cues indicating their own smaller scale.*

With that being said, if this article were rewritten to address these issues I think the main contribution—of moving beyond Stroop(-like) Tests and IATs in establishing the principles of CMT and certain theories of embodied cognition to more complicated models of how bodies move through space—is an important one and worth publishing.

Response: Thank you for your suggestion. We have made the corresponding supplement. In addition, we also express our gratitude for your meticulous guidance. The modifications are as follows:

*Finally, it should be noted that while the current investigation has predominantly employed behavioral experimental paradigms, it remains limited in its engagement with cognitive neuroscience frameworks. Future studies could incorporate more embodied interaction tasks combined with relevant physiological measures, providing multidimensional empirical evidence for subsequent research.*

*This study illuminates the unconscious psychological relationship underlying size-space metaphors in moral cognition within the framework of embodies cognition, with a specific focus on how spatial environments may shape ethical decision-making (from the variants of Stroop and IAT to the specific physical experience model).*

*This study not only provides novel perspectives on moral reasoning, but also offers more empirical insights to advance the theoretical framework of embodied cognition.*
